# YvcK, a protein required for cell wall integrity and optimal carbon source utilization, binds uridine diphosphate-sugars

**DOI:** 10.1038/s41598-017-04064-2

**Published:** 2017-06-23

**Authors:** Elodie Foulquier, Anne Galinier

**Affiliations:** Laboratoire de Chimie Bactérienne, CNRS - Aix Marseille Univ, IMM, 31 Chemin Joseph Aiguier, 13402 Marseille, Cedex 20 France

## Abstract

In *Bacillus subtilis*, *Listeria monocytogenes* and in two *Mycobacteria*, it was previously shown that *yvcK* is a gene required for normal cell shape, for optimal carbon source utilization and for virulence of pathogenic bacteria. Here we report that the *B. subtilis* protein YvcK binds to Uridine diphosphate-sugars like Uridine diphosphate-Glucose (UDP-Glc) and Uridine diphosphate-*N*-acetylglucosamine (UDP-GlcNAc) *in vitro*. Using the crystal structure of *Bacillus halodurans* YvcK, we identified residues involved in this interaction. We tested the effect of point mutations affecting the ability of YvcK to bind UDP-sugars on *B. subtilis* physiology and on cell size. Indeed, it was shown that UDP-Glc serves as a metabolic signal to regulate *B. subtilis* cell size. Interestingly, we observed that, whereas a *yvcK* deletion results in the formation of unusually large cells, inactivation of YvcK UDP-sugar binding site does not affect cell length. However, these point mutations result in an increased sensitivity to bacitracin, an antibiotic which targets peptidoglycan synthesis. We thus propose that UDP-GlcNAc, a precursor of peptidoglycan, could be a good physiological ligand candidate of YvcK.

## Introduction

In bacteria, several cellular processes like cell division, cell size and morphogenesis, are tightly coordinated with metabolism^1^. For example, during growth in a poor medium, bacterial cell size can be reduced to half the length in comparison to growth in a rich medium^2^. The *Bacillus subtilis* YvcK protein might play a role in the maintenance of bacterial shape in a nutrient dependent manner^3–5^.

Indeed, in *B. subtilis*, YvcK was previously shown to be unnecessary when bacteria were grown in glycolytic carbon sources but essential for growth in Krebs cycle intermediates and in carbon sources metabolized via the pentose phosphate pathway^3^. In these carbon sources, *yvcK* mutant cells display morphologic abnormalities, including bulging cells before lysis. The cell wall, with its major structural component the peptidoglycan (PG), forms the protective barrier that maintains the integrity and the shape of the cell. Its expansion is orchestrated by cytoskeletal proteins^6^. By deconvolution fluorescence microscopy, it was observed that YvcK has a helix-like cellular localization as it was previously described for MreB, the key actin-like cytoskeletal component in rod-shaped bacteria^4, 7^. The use of high resolution fluorescence microscopy has revealed that MreB forms motile patches whose movements are dependent on the cell wall synthesis machinery^8, 9^. The *mreB* mutant exhibits a characteristic bulging phenotype and PBP1, the major penicillin-binding protein implicated in PG synthesis, is mislocalized^10^. Amazingly, overproduction of YvcK restores proper PBP1 localization and rescues the morphology defects of cells lacking MreB. Moreover, PBP1 is also delocalized in a *yvcK* mutant grown in minimal medium supplemented with gluconate and the deletion of the PBP1 encoding gene restores the growth of a *yvcK* mutant in this restrictive medium^4^. Thus, all these results suggest that, though its mechanism of action is still unknown, YvcK is required for proper PBP1 localization and normal cell wall biosynthesis or integrity in *B. subtilis*. Also, a *yvcK* mutant is more sensitive than the WT strain to bacitracin, an antibiotic that inhibits cell wall synthesis by binding to undecaprenyl-pyrophosphate, the phosphorylated form of the carrier molecule for PG precursors^11, 12^.

The YvcK protein is present in a wide variety of bacteria and its role has also been investigated in pathogens. In two mycobacterial species, *M. smegmatis* and *M. tuberculosis*, the deletion of *cuvA*, the gene encoding the YvcK orthologue, leads to changes in nutrient uptake and/or metabolism that affect cell wall structure, morphology and bacterial virulence^5^. Strains lacking *cuvA* display growth defects in several conditions using different carbon sources. These strains also have morphological defects and a hypersensitivity to several β-lactam antibiotics that inhibit synthesis of PG. In both mycobacterial species, CuvA localizes only to the growing cell pole, the site of PG synthesis^5^. In *Listeria monocytogenes*, it has recently been shown that YvcK is also required for cell wall stress responses, growth in glycerol medium, cytosolic survival and virulence^13^.

To gain information on YvcK cellular function(s), a transposon mutagenesis approach was carried out in order to identify suppressors that rescue the *B. subtilis* Δ*yvcK* mutant and the *M. tuberculosis* Δ*cuvA* mutant^3, 5^. This approach was not very informative for *B. subtilis* despite the isolation of revertants with insertion in *yfnI* ^3^, a gene encoding a lipoteichoic acid synthase involved in cell wall synthesis^14^. However, for *M. tuberculosis*, the isolation of transductants with insertions in *pbpA* and *rodA*, two genes involved in PG synthesis and cell shape control, indicates a link between CuvA and cell wall synthesis and morphology^5^. This result is reminiscent of the rescue of the *B. subtilis yvcK* mutant by deletion of the gene encoding PBP1^4^. In addition CuvA specifically localizes to the growing cell pole, where the peptidoglycan synthesis occurs in mycobacteria^5^, whereas YvcK is localized as a helical-like pattern along the length of the cell^4^, where the PG synthesis machinery is evenly dispersed allowing a cylindrical elongation of the rod-shaped Bacillus cells^8, 9, 15, 16^.

This YvcK/CuvA protein belongs to the UPF0052 uncharacterized protein family and its biochemical properties are unknown. It harbors a Rossmann fold, a structural motif that is commonly observed in enzymes regulated by dinucleotide coenzymes such as FAD and NAD(P)^17^. No binding of mononucleotides like GTP, GDP or ATP, ADP to *B. subtilis* YvcK was detected but the crystal structure of *Bacillus halodurans* YvcK was obtained when associated with NAD^18^. In this paper, we have undertaken the biochemical characterization of YvcK from *B. subtilis* and we have shown that this protein is able to bind UDP-sugars like UDP-GlcNAc and UDP-Glc *in vitro*. We have also identified the residues involved in UDP-sugar binding. We observe that inactivation of the UDP-sugar binding site does not affect cell size and growth on non-permissive medium. By contrast, mutants where the ability of YvcK to bind UDP-sugar is affected, have an increased sensitivity to bacitracin suggesting that UDP-GlcNAc, a key precursor of PG, could be the physiological ligand of YvcK.

## Results and Discussion

### YvcK is a UDP-sugar binding protein

To determine its biochemical properties, the YvcK protein was over-expressed, purified and its ability to bind dinucleotides was analyzed. However, we did not detect any binding with micromolar concentrations of FAD or NAD(P) (reduced and oxidized forms) with all the techniques tested. In an analysis of *Lactobacillus rhamnosus* genome predicting novel glycosyltransferases, YvcK was proposed as a putative one^19^. Glycosyltransferases are enzymes which transfer sugar moieties from an activated donor to a specific substrate. Since a *yvcK* mutant has an increased sensitivity to bacitracin^12^, we tested the binding of YvcK with two UDP-sugars, UDP-Glc and UDP-GlcNAc, that are cell wall precursor components.

**Figure 1:**
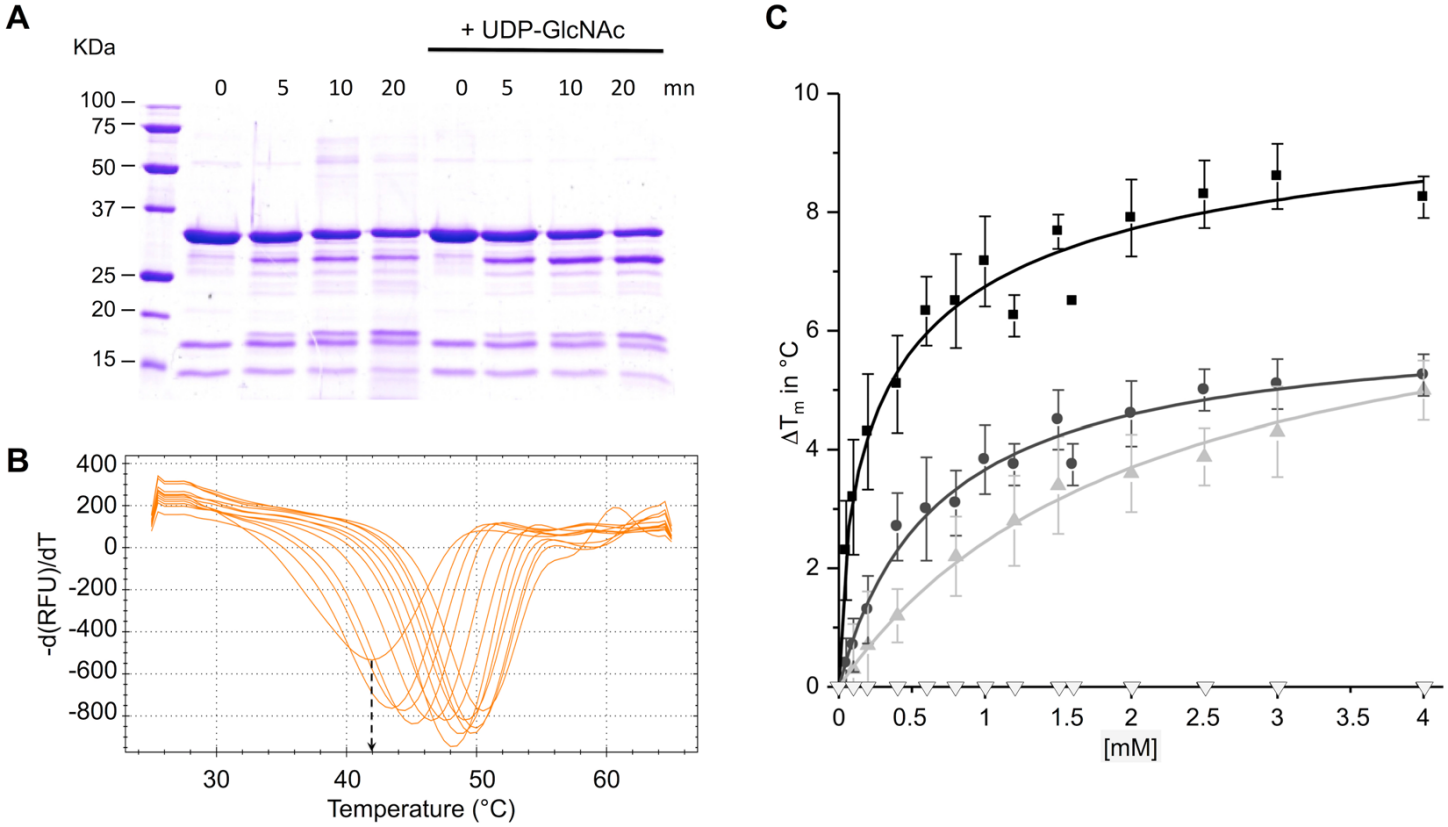
YvcK binds to UDP, UDP-Glc and UDP-GlcNAc. (**A**) Coomassie-stained SDS-PAGE of YvcK partial proteolysis profile. YvcK was incubated with Trypsin (Promega) in the absence or in the presence of 1 mM UDP-GlcNAc for 0, 5, 10 or 20 min at 37 °C. The digestion profiles were assessed by electrophoresis in 12.5% SDS-PAGE. Full-length gel is presented in Supplementary data. (**B**) Thermal Shift Assay (TSA) in the presence of increasing concentrations of UDP-GlcNAc. YvcK melting profiles were monitored in the presence of increasing concentration of UDP-GlcNAc (0 to 4 mM). One curve corresponds to data obtained for one concentration of UDP-GlcNAc. The melting temperature of the protein (T_m_) is obtained at the midpoint of the each melting curve and corresponds to the minimum of the negative derivative curves (see the arrow that indicates the T_m_ of YvcK in the absence of UDP-GlcNAc). The T_m_ is an indicator of protein stability and is increased by the addition of UDP-GlcNAc (T_m_ = 42 °C in the absence of UDP-GlcNAc and T_m_ = 50.5 °C in the presence of 4 mM UDP-GlcNAc). (**C**) TSA results for the binding of UDP, UDP-Glc, UDP-GlcNAc and Glc-NAc. Assays were performed in the presence of increasing concentrations of ligands (0 to 4 mM). The difference of temperature (the shift of T_m_ induced by the presence of ligand) was plotted against the concentration of UDP (black circle), UDP-Glc (grey triangle), UDP-GlcNAc (black square) and Glc-NAc (white triangle). All the curves correspond to the average of data from at least 3 independent experiments and the standard deviations are representated by the error bars. Curve fitting was performed by using Microcal Origin 5.0 software.

For this purpose, we assessed YvcK for sensitivity to limited protease digestion *in vitro*. As shown in Fig. 1A, the presence of UDP-GlcNAc weakly modifies YvcK trypsin sensitivity. Indeed, after 20 min of digestion, the band at around 35 kDa corresponding to YvcK is partially degraded to four main bands. In the presence of UDP-GlcNAc, YvcK is still partially degraded to four main bands but the band of 30 kDa is more intense and thus seems to be more resistant to the trypsin cleavage. This result suggests a direct binding of UDP-GlcNAc to YvcK. This binding probably induces conformational changes of YvcK that result in the protection from trypsin cleavage. A similar result was obtained with UDP-Glc (data not shown). To confirm and quantify these interactions we carried out Thermal Shift Assay (TSA); this approach was successfully used to investigate protein-ligand interactions^20^ and to determine apparent *K*_D_^21^. By monitoring SYPRO^®^ Orange dye fluorescence in microplates using a thermal cycler, we have quantified the effects of ligands on YvcK melting temperature (Fig. 1B). We found that UDP-GlcNAc and UDP-Glc raise the melting temperature (T_m_) of YvcK in a concentration-dependent manner (Fig. 1C). Moreover, the observed increases of T_m_ (of up to 8 °C) indicate that binding of UDP-sugars significantly stabilizes the structure of YvcK (Fig. 1B). In addition, we observed that YvcK possesses a five-fold higher affinity for the UDP-GlcNAc (apparent *K*_D_ = 0.41 ± 0.24 mM) than UDP-Glc (apparent *K*_D_ = 2.11 ± 0.65 mM). We also analyzed the effect of the two moieties of UDP-GlcNAc separately; namely UDP and GlcNAc on the T_m_ of YvcK. Whereas UDP was able to bind to YvcK, no binding of GlcNAc was detected (Fig. 1C). This observation suggests that the presence of the UDP moiety in the ligand is necessary for the interaction with YvcK. Moreover, UDP alone binds YvcK with an affinity lower than that of the two UDP-sugars tested (apparent *K*_D_ = 6.21 ± 0.62 mM). This result indicates that the presence of a sugar to the UDP moiety increases the affinity (Fig. 1C) and suggests that YvcK may probably bind other UDP-sugars.

YvcK binds UDP-Glc and UDP-GlcNAc with relatively low apparent affinities and we can wonder if the binding of these two UDP-sugars has any significance *in vivo*. Up to now, their intracellular concentrations were not determined in *B. subtilis* but we can suppose that they are likely high in actively growing cells because bacteria use large amounts of these metabolites in their peptidoglycan, teichoic acids and lipopolysaccharides biosynthesis. For example in *Escherichia coli*, the intracellular concentration of UDP-GlcNAc was determined and, in the conditions tested *i.e*. exponential phase growth in LB medium, the value obtained was approximately 0.43 mM^22^. This value belongs to the same scale to that of the apparent affinity of *B. subtilis* YvcK for this metabolite. Consequently, if the concentration of UDP-GlcNAc in *B. subtilis* cells is similar to that in *E. coli* cells, it is conceivable that UDP-GlcNAc could bind to YvcK *in vivo*.

### A *yvcK* deletion affects cell length

In *B. subtilis*, UDP-Glc serves as a metabolic signal to regulate cell size in response to enhanced carbon availability by a well-characterized mechanism mediated by UgtP, an enzyme involved in the synthesis of glycolipids and the anchoring of lipoteichoic acids^23–25^. Under rich conditions, UgtP binds to UDP-Glc and is concentrated at midcell and inhibits FtsZ in rapidly growing cells, causing a delay of cell division. Under poor growth conditions, when the availability of UDP-Glc is low, UgtP is free and appears to be sequestered in randomly distributed foci where it is presumably unable to modulate FtsZ assembly. This change of UgtP localization seems to be controlled by the availability of UDP-Glc, a substrate of UgtP; a deletion of *ugtP* gene results in reduced cell size, in particular under rich growth conditions^25, 26^. Because YvcK function is in relation with cell wall synthesis (or integrity) and it also binds UDP-Glc *in vitro*, we wonder if it participates in cell size regulation. For this purpose, we constructed a non-polar markerless *yvcK* deletion mutant and checked that the genes downstream of *yvcK* were correctly expressed. Indeed it was previously observed that cells deleted for *yvcL*, the gene just downstream of *yvcK*, are longer than WT cells^27^.

**Figure 2:**
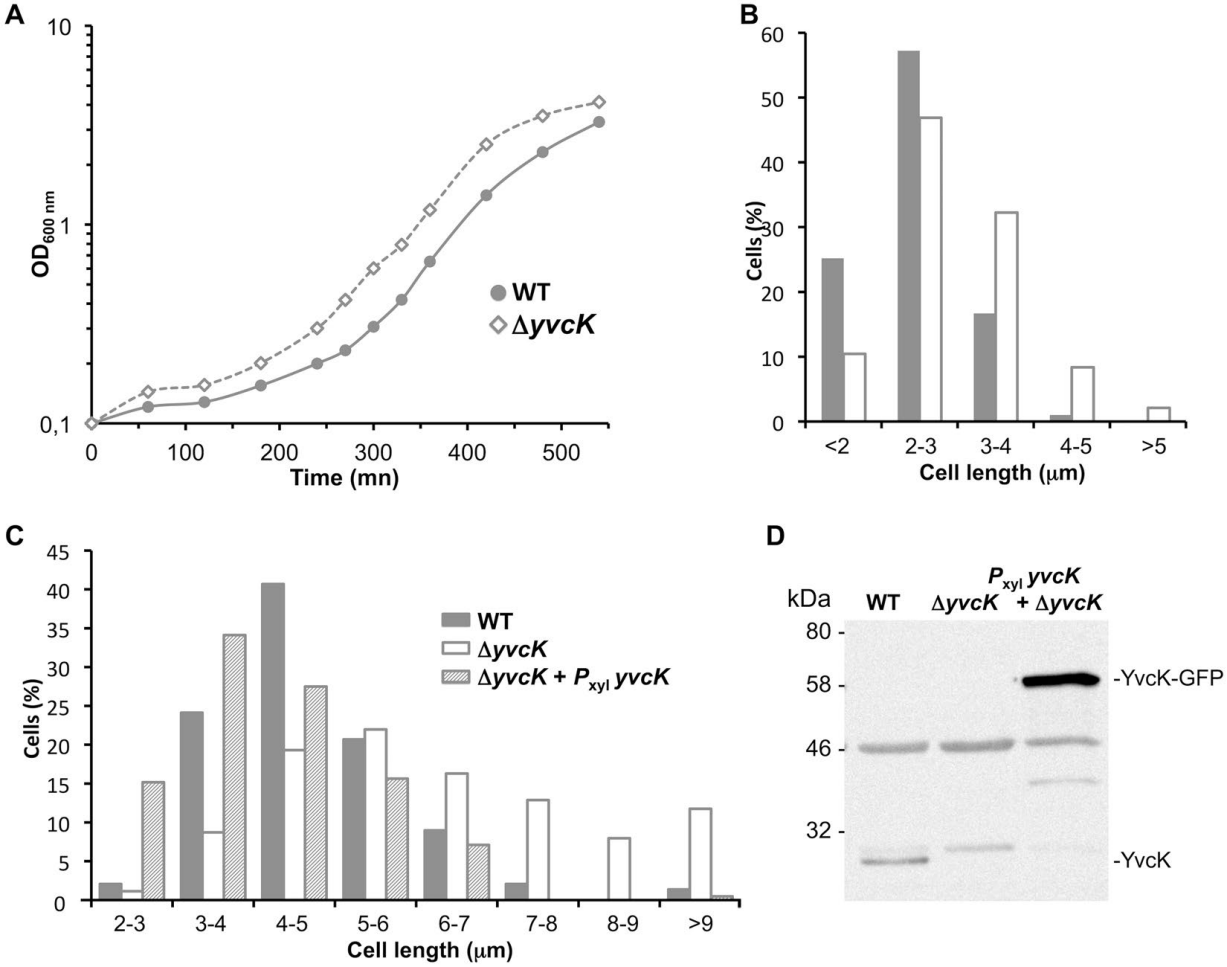
YvcK regulates cell length. (**A**) Growth curve of WT strain (168) and Δ*yvcK* mutant strain (SG471) on minimal medium with sorbitol as sole carbon source. (**B**) Histogram of cell lengths for 168 and SG471 strains collected at OD_600_ = 0.4: WT (n = 306), Δ*yvcK* strain (n = 335). (**C**) Histogram of cell lengths for WT strain (168), Δ*yvcK* strain (SG471) and Δ*yvcK* strain with *P*_xyl_ *yvcK* at *amyE* locus (SG517). These three strains were grown in LB medium in the presence of xylose and collected at OD_600_ = 0.4: WT (n = 319), Δ*yvcK* strain (n = 256), Δ*yvcK, P*_xyl_ *yvcK* strain (n = 301). (**D**) Analysis of YvcK production from crude extracts of WT strain (168), Δ*yvcK* strain (SG471) and Δ*yvcK* strain with *P*_xyl_ *yvcK* at *amyE* locus (SG517) by Western blotting. Bacteria were collected at OD_600_ = 0.4, lyzed and 10 μl of cell extracts were run on a 12.5% SDS-PAGE and transferred to nitrocellulose membrane by electroblotting. The blot membrane was exposed for each time set in interval time and accumulates images. Full-length blot is presented in Supplementary data.

For these experiments concerning the analysis of the Δ*yvcK* cell size, we chose experimental conditions in which *yvcK* mutant strain has a growth rate comparable to that of the WT strain; we thus excluded media where the sole carbon source is a Krebs cycle intermediate or a substrate of pentose phosphate pathway^3^. As it was previously done for the studies of *ugtP* mutant^25, 26^, we used two growth media. In the first one, we used minimal medium supplemented with sorbitol (glucitol) as sole carbon source; in these poor growth conditions the UDP-Glc intracellular concentration is supposed to be low and the cells short. The second medium used is LB; in these rich growth conditions the UDP-Glc intracellular concentration is supposed to be high, the cells large and the effect of a *ugtP* deletion was amplified. As expected, the *yvcK* mutant strain growth in minimal medium containing sorbitol is similar to that of the WT strain indicating that the growth rate is not impaired (Fig. 2A) and ref. 3. However, the *yvcK* mutant cells (average size about 2.9 μm) seem larger than the WT cells (average size about 2.4 μm) (Fig. 2B). This size increase is weak but, in this poor growth condition, cells are short^25, 26^. To confirm that this increase of cell length is really due to the absence of YvcK, we constructed a new strain by introducing a copy of *yvcK* allele fused to *gfp* under the control of *P*_xyl_ promoter in the *yvcK* mutant strain. Then we analyzed the length of cells grown in the presence of xylose, in a LB rich medium where the bacterial cell size is large^25, 26^. We showed that, as observed in minimal medium, in LB medium the *yvcK* mutant cells are larger than the WT cells (average sizes about 6.3 μm for Δ*yvcK* cells vs 4.7 μm for WT cells) and this cell size increase is more obvious than in poor medium (Fig. 2C). Furthermore, in presence of xylose, the *yvcK* mutant cells overexpressing *yvcK-gfp* gene fusion under the control of *P*_xyl_, are smaller than the WT cells (about 0.6 μm of difference; average size about 4.1 μm for cells overproducing YvcK-GFP) (Fig. 2C and D). We conclude that YvcK regulates cell length and its gene deletion induces larger cells.

It was recently shown that the availability of PG precursors plays an essential role in determining cellular dimensions across diverse species of bacteria^28^. Furthermore, another study showed that knockdowns of genes encoding PG biosynthesis enzymes also produced elongated *B. subtilis* cells^29^. The cell size increase of a *yvcK* mutant could be due to an alteration of PG synthesis but it can also have other causes and could also be related to the UDP-Glc and UgtP pathway.

### A *yvcK* deletion enhances the effect of deletions of *ugtP* and *gtaB* genes

**Figure 3:**
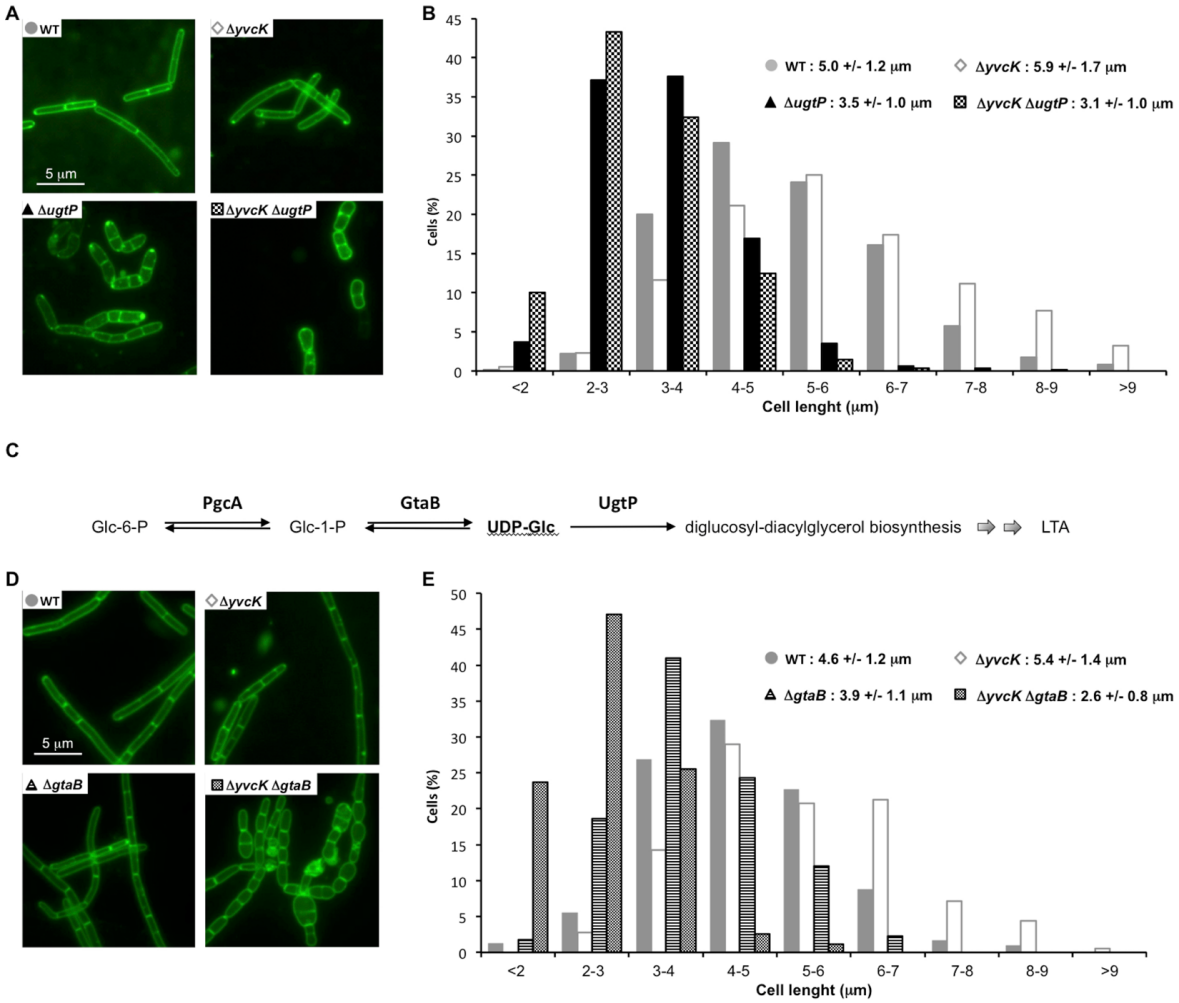
A *yvcK* deletion enhances the effect of deletions of *ugtP* and *gtaB* genes. Strains were grown on LB medium until OD_600_ = 0.4. (**A**) Micrographs of WT strain (168) and mutant strains Δ*yvcK* strain (SG471), Δ*ugtP* strain (SG571), Δ*ugtP* Δ*yvcK* strain (SG561). Membranes were stained with FM1-43. (**B**) Histogram of cell lengths for WT strain (n = 1032), Δ*yvcK* strain (n = 611), Δ*ugtP* strain (n = 970) and Δ*ugtP* Δ*yvcK* strain (n = 626). (**C**) Simplified scheme of the first steps of the glycolipid metabolism. Enzymes involved in this pathways are PgcA: Phosphoglucomutase, GtaB: UTP-glucose-1-phosphate uridylyltransferase, UgtP: Processive diacylglycerol beta-glucosyltransferase. (**D**) Micrographs of WT strain (168) and mutant strains Δ*yvcK* strain (SG471), Δ*gtaB* strain (DKE35670), Δ*gtaB* Δ*yvcK* strain (SG562). Membranes were stained with FM1-43. E- Histogram of cell lengths for WT strain (n = 390), Δ*yvcK* strain (n = 183), Δ*gtaB* strain (n = 355) and Δ*gtaB* Δ*yvcK* strain (n = 270).

The two proteins YvcK and UgtP are both able to bind UDP-Glc but, contrarily to a *ugtP* deletion that results in the formation of unusually small cells^25^, we showed here that a deletion of *yvcK* produces large daughter cells. To study these antagonist effects, we decided to construct an *ugtP yvcK* double mutant in order to measure its cell length and compare it to that of a WT, a *ugtP* mutant and a *yvcK* mutant in a rich LB medium (Fig. 3A and B). Remarkably, the cell length distribution of the *ugtP yvcK* double mutant resembles that of the *ugtP* mutant but cells of the double mutant are even smaller than that of the single mutant (about 3.1 μm vs 3.5 μm) as if a *yvcK* deletion enhances the effect of a *ugtP* deletion. This result is unexpected because it is the opposite effect of that obtained with the single deletion *yvcK* mutant that causes elongated cells.

UgtP is not only an FtsZ inhibitor; it is also the third and the last enzyme of the glycolipid biosynthesis pathway. In this pathway, UDP-Glc is generated from Glc-6-P by two reversible steps, catalyzed by the two upstream enzymes PgcA or GtaB (Fig. 3C). A deletion of *ugtP*, *gtaB* or *pgcA* genes causes a similar cell length phenotype, i.e. reduced cell size when bacteria were grown in rich medium^25^. In order, to test whether the effect of a *yvcK* deletion associated with a deletion of a gene encoding another enzyme of the glycolipid biosynthesis pathway (in conditions where UDP-Glc synthesis is altered), we constructed a *gtaB yvcK* double mutant and analyzed its cell size in LB rich conditions (Fig. 3D and E). We observed that, as expected the *gtaB* simple mutant cells are smaller than WT cells but larger than that of the *ugtP* mutant. In addition, the cell length distribution showed that the *gtaB yvcK* mutant cells are smaller than the *gtaB* mutant cells and similar to that of the *ugtP yvcK* mutant. Indeed, for both double mutants, the average cell size is about 1.9 or 2 μm shorter than that of the WT cells (Fig. 3B and E). These data suggest that a *yvcK* deletion enhances the effect of deletions of genes encoding an enzyme of the glycolipid biosynthesis pathway, *ugtP* or *gtaB*, to obtain critically short cells.

### Characterization of the YvcK residues involved in the binding of UDP-sugars

**Figure 4:**
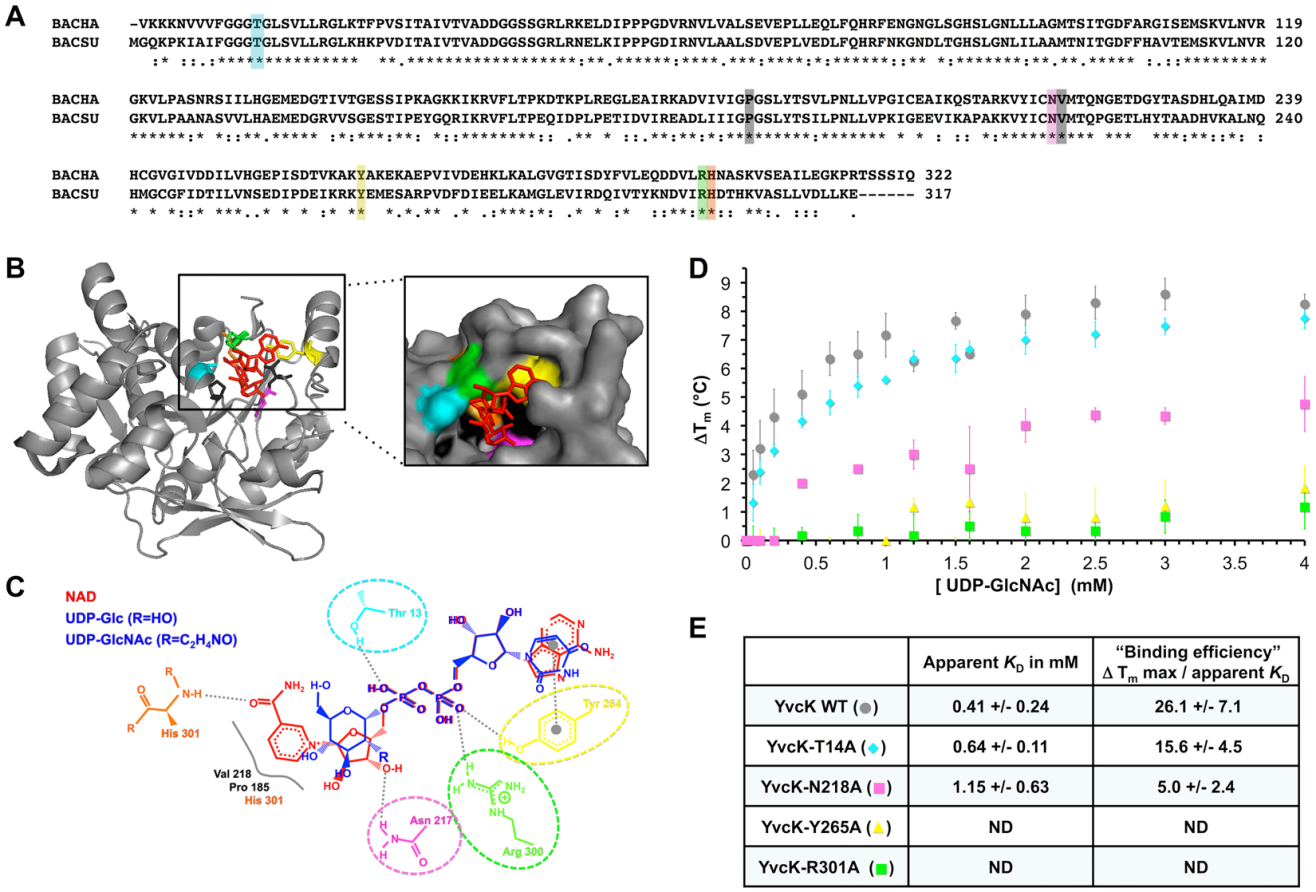
Identification of residues involved in UDP-sugars binding. (**A**) Sequence alignment of YvcK from *B. subtilis* and from *B. halodurans*. The highlighted amino acids are those found to interact with NAD in the crystal structure. (**B**) Crystal structure of a monomer of *B. halodurans* YvcK in complex with NAD (PDB ID: 2O2Z). The colored residues of YvcK were found to interact with NAD. The figure was realized with PyMOL software. (**C**) Identification of YvcK residues susceptible to interact with UDP-sugars. This scheme generated by PoseView software represents the residues of *B. halodurans* YvcK that interact with NAD. A molecule of UDP-Glc or UDP-GlcNAc was superposed to NAD and residues of YvcK that could interact with UDP-Glc or UDP-GlcNAc are encircled. (**D**) TSA results for the binding of UDP-GlcNAc to *B. subtilis* YvcK modified proteins. Four YvcK residues found to interact with NAD in the crystal structure were replaced by Ala to generate YvcK-T14A, YvcK-N218A, YvcK-Y265A and YvcK-R301A. The proteins were purified and TSA was performed in the presence of increasing concentrations of UDP-GlcNAc (0 to 4 mM). The difference of temperature was plotted against the concentration of UDP-GlcNAc for YvcK (grey circle) and each modified protein: YvcK-T14A (cyan diamond), YvcK-N218A (pink square), YvcK-Y265A (yellow triangle) and YvcK-R301A (green square). All the values correspond to the average of data from at least 3 independent experiments and the standard deviations are representated by the error bars. (**E**) Kinetic parameters for the binding of UDP-GlcNAc to *B. subtilis* YvcK modified proteins from TSA experiments. The apparent *K*_D_, the Δ T_m_ max and the standard deviations were calculated using Microcal Origin 5.0 software. ND indicates that the binding of UDP-GlcNAc to YvcK and the Δ T_m_ values are too weak to calculate an apparent *K*_D_.

To gain more information on the mysterious role of YvcK on cell size, we analyzed mutants of *yvcK* encoding YvcK protein affected in its ability to UDP-sugar. YvcK from *B. haludurans* possesses 63% identity and 86% similarity with YvcK from *B. subtilis* (Fig. 4A). The structure of *B. haludurans* YvcK was previously obtained in complex with NAD (Fig. 4B). The residues found to interact with the dinucleotide in the crystal structure are conserved in *B. subtilis* YvcK (Fig. 4A). The structures of the three molecules NAD, UDP-Glc and UDP-GlcNAc possess similarities; the 2 phosphates and a D-ribose can be overlaid (Fig. 4C). We can thus speculate that some residues found to interact with NAD in the YvcK structure, Thr13, Asn217, Tyr264 and Arg300 (numbering from *B. halodurans*), could interact with UDP-Glc (+/−NAc). To test this hypothesis, we replaced these four amino acids by Ala in *B. subtilis* YvcK protein. The corresponding proteins, YvcK-T14A, YvcK-N218A, YvcK-Y265A and YvcK-R301A, were overexpressed and purified as for the wild-type enzyme. Then, their proteolysis pattern was checked; for each mutant protein, it was similar to that of the WT protein in the absence of UDP-GlcNAc suggesting that the overall structure seemed to be unaffected by the introduced mutations (data not shown). Hence, their ability to bind UDP-GlcNAc was tested by TSA. In the absence of nucleotide, the four mutant proteins have a T_m_ around 40+/− 2 °C (T_m_ of the WT protein is about 42 °C see Fig. 1B) confirming that the global structure is not affected by the mutations. We found that the ability to bind UDP-GlcNAc is affected at different levels in the four modified proteins (Fig. 4D and E). In particular, whereas the replacement of Thr14 by an Ala, and at a less extent that of Asn218 by an Ala, slightly affects the binding of UDP-GlcNAc, the replacement of Tyr265 or Arg301 by Ala strongly affects this binding. These results show that some residues found to interact with NAD in the structure interact with UDP-sugars *in vitro*.

### Point mutations of the UDP-sugar binding site do not affect neither cell size in rich medium nor growth and morphology in minimal medium supplemented with gluconate

**Figure 5:**
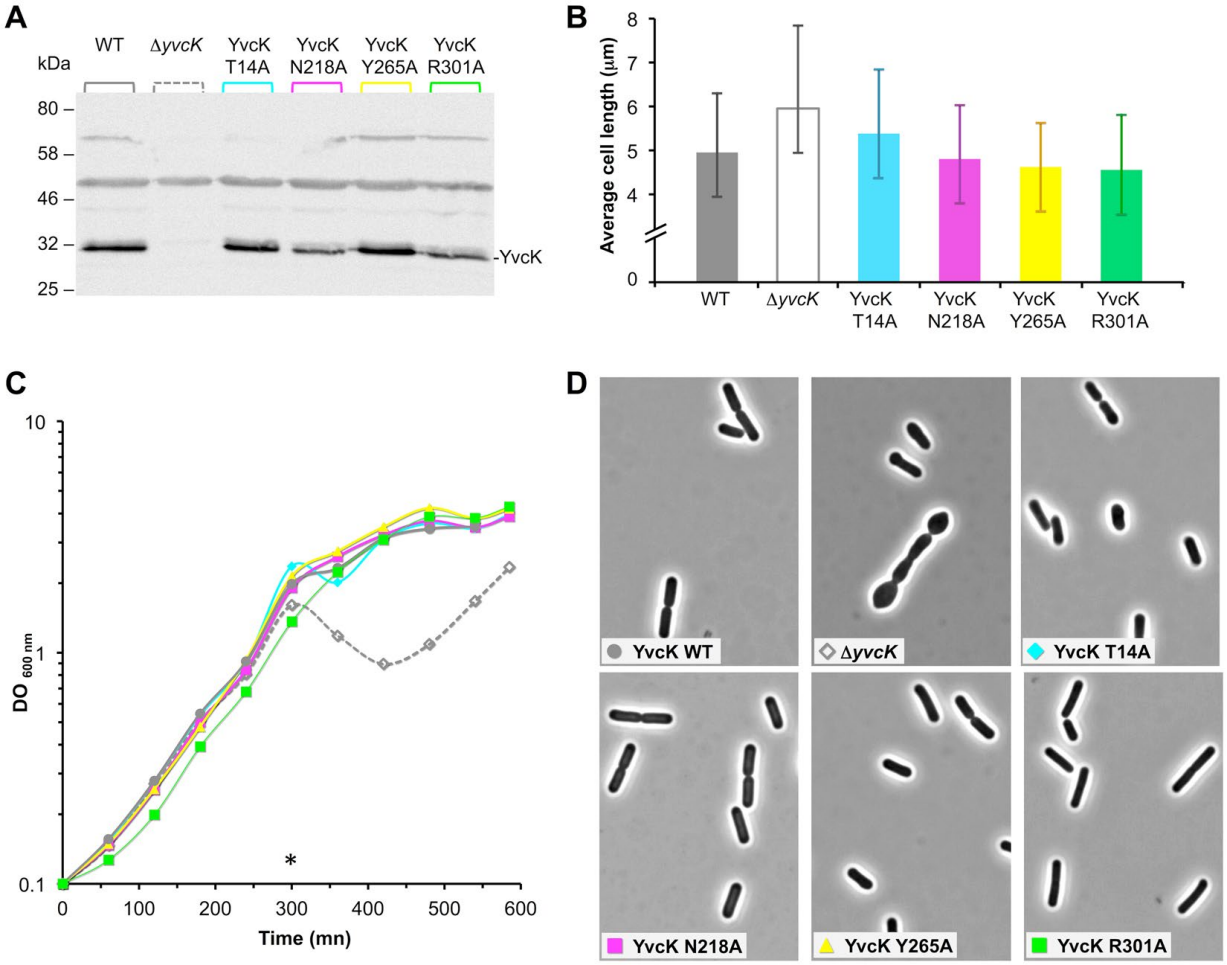
Mutations of residues interacting with UDP-sugars do not affect cell length and bacterial growth on minimal medium containing gluconate as sole carbon source. (**A**) Analysis of YvcK production by Western blotting. WT strain (168), Δ*yvcK* strain (SG471) and strains producing YvcK-T14A (SG520), YvcK-N218A (SG521), YvcK-Y265A (SG522) and YvcK-R301A (SG523) were grown at 37 °C in 20 ml of LB medium and collected at OD_600_ = 1. 16 μl of cell extracts were run on a 12.5% SDS-PAGE and transferred to nitrocellulose membrane by electroblotting. The blot membrane was read by the LAS4000 mini Imager. Full-length blot is presented in Supplementary data. (**B**) Bar graph of cell length averages for WT strain (168), Δ*yvcK* strain (SG471) and strains producing YvcK-T14A (SG520), YvcK-N218A (SG521), YvcK-Y265A (SG522) and YvcK-R301A (SG523). Bacteria were grown on LB medium until OD_600_ = 0.4 and analyzed by microscopy. The data were obtained from four independent experiments and the standard deviations are representated by the error bars. (**C**) Effect of *yvcK* point mutations on *B. subtilis* growth on gluconate as sole carbon source. WT strain (168; grey circle), Δ*yvcK* strain (SG471*;* empty grey diamond) and strains producing YvcK-T14A (SG520; cyan diamond), YvcK-N218A (SG521; YvcK pink square), YvcK-Y265A (SG522; yellow triangle) and YvcK-R301A (SG523; green square) were grown overnight on LB medium with 1% glucose. After centrifugation, cells were grown in CE-gluconate liquid medium at 37 °C supplemented with 0.5% xylose. (**D**) Effect of *yvcK* point mutations on cell morphology during growth on gluconate as sole carbon source. Cells were grown in minimal medium supplemented with gluconate at 37 °C and collected after 300 mn of growth. Their morphology was analyzed by microscopy: see micrographs of WT strain (168; grey circle), Δ*yvcK* strain (SG471*;* empty grey diamond) and strains producing YvcK-T14A (SG520; cyan diamond), YvcK-N218A (SG521; pink square), YvcK-Y265A (SG522; yellow triangle) and YvcK-R301A (SG523; green square).

Since YvcK regulates cell length, we investigated whether *yvcK* point mutant cells are larger than WT cells when its ability to bind UDP-Glc is affected by point mutations. For this purpose, we constructed non-polar markerless strains expressing the mutated alleles of *yvcK* at a physiological level. After growth of these strains in LB medium, we carried out western blot analysis in order to verify the expression level of each mutant protein (YvcK-T14A, YvcK-N218A, YvcK-Y265A and YvcK-R301A). We observed that, as expected, the growth of the mutant strains was normal and similar to WT strain (data not shown) and that each mutant protein was expressed (Fig. 5A). Then, we analyzed the cell size of these strains grown in LB medium (Fig. 5B). However, surprisingly, when we measured the cell size and in particular the average length of each strain, we did not find any obvious correlation between the increase of cell length and the inability of the corresponding mutant YvcK protein to bind UDP-sugars (Fig. 5B vs Fig. 4D and E). Indeed cells expressing the proteins YvcK-Y265A or YvcK-R301A, whose ability to bind UDP-sugars is almost lost, have an average length similar to that of the strains expressing WT YvcK or YvcK-N218A, whose ability to bind UDP-sugars is moderately affected. Cells expressing the YvcK-T14A protein, whose ability to bind UDP-sugars is slightly affected, have an average cell length between that WT and *yvcK* mutant strains (about 5.4 μm vs 5.0 μm and 6.0 μm, respectively).

We also tested the effect of these four substitutions on bacterial growth in minimal medium containing gluconate as source of carbon (Fig. 4C). In these growth conditions, a strain deleted for *yvcK* has a growth defect^3, 4^. All the strains expressing the YvcK modified proteins have a normal growth and a normal cell shape, even for the strains expressing the proteins YvcK-Y265A or YvcK-R301A, whose ability to bind UDP-sugars is strongly disturbed (Fig. 4C and D). By contrast, as expected, the strain deleted for *yvcK* cannot grow normally in this medium. These data indicate that the ability of YvcK to bind UDP-sugars is not crucial for *B. subtilis* growth and morphology in minimal medium containing gluconate.

### Point mutations of the UDP-sugar binding site affect bacitracin sensitivity

We previously observed that a *yvcK* mutant has an increased bacitracin sensitivity in comparison with the WT strain^12^. This antibiotic inhibits cell wall biosynthesis by interfering with the dephosphorylation of C55-isoprenyl pyrophosphate, a membrane carrier molecule that transports the building-blocks of the PG^11, 12^. Because UDP-sugars and particularly UDP-GlcNAc are key precursors of PG, we tested the effect of point mutations of the YvcK UDP-sugar binding site on bacitracin sensitivity. Thus, for each mutant strain, we determined the bacitracin concentration needed to inhibit half of the bacterial growth (Table 1). We observed that strains expressing the YvcK-Y265A or YvcK-R301A protein, whose ability to bind UDP-GlcNAc is almost lost *in vitro* (Fig. 4D), have IC_50_ values similar to the *yvcK* mutant strain (about 135 μg/ml and 172 μg/ml for strains producing YvcK-Y265A or YvcK-R301A protein respectively and 139 μg/ml for the *yvcK* mutant strain). By contrast, strains expressing the YvcK-T14A or YvcK-N218A protein, whose ability to bind UDP-sugars is more weakly affected (Fig. 4D and E), have IC_50_ values that tend towards that of the WT strain (about 282 μg/ml and 261 μg/ml for strains producing YvcK-T14A or YvcK-N218A protein respectively and 368 μg/ml for the WT strain). These data show a correlation between the increase of bacitracin sensitivity and the alteration of the corresponding mutant YvcK protein to bind UDP-GlcNAc and demonstrate a physiological role for the residues involved in UDP-sugars binding. UDP-GlcNAc being a key precursor of PG and bacitracin targeting the synthesis of PG, we propose that this UDP-sugar could be a good candidate to be the physiological ligand of YvcK.

**Table 1:** Effect of *yvcK* point mutations on antibiotic resistance.

Strains	Genotypes	Bacitracin IC_50_ in μg/ml
168	*trpC2*	368 ± 27
SG471	*trpC2, yvcK*Δ*1*	139 ± 18
SG520	*trpC2, yvcK T14A*	282 ± 22
SG521	*trpC2, yvcK N218A*	261 ± 63
SG522	*trpC2, yvcK Y265A*	135 ± 30
SG523	*trpC2, yvcK R301A*	172 ± 39

Bacitracin resistance of WT strain (168) and Δ*yvcK* strain (SG471) used as controls and of strains producing YvcK-T14A (SG520), YvcK-N218A (SG521), YvcK-Y265A (SG522) and YvcK-R301A (SG523) was determined in 96-well microplates. At the end of the incubation period, OD_600 nm_ was monitored using a Tecan microplate reader. Results are expressed as average of IC_50_ values (i.e. bacitracin concentrations inhibiting 50% of bacterial growth) obtained in at least 3 independent experiments ± standard deviations.

Furthermore, because alteration of UDP-sugar binding site does not have any influence on growth in gluconate medium and no obvious effect on cell size but affects sensitivity to bacitracin, this implies that YvcK has at least two cellular roles. One, independent of its ability to bind UDP-sugars, is involved in the regulation of cell size and of bacterial growth and morphology where the sole carbon source available is a Krebs cycle intermediate or a substrate of the pentose phosphate pathway. Another one, dependent on its ability to bind UDP-sugars, is related to bacitracin sensitivity and potentially to PG synthesis. This multifunctional aspect of YvcK was already mentioned in previous studies. Indeed, YvcK was found to be phosphorylated in *B. subtilis*, in *M. tuberculosis* and *in L. monocytogenes* ^5, 12, 13, 30^. The role of this phosphorylation was analyzed by mutations of the phosphorylated residue(s) in YvcK and inactivation of the corresponding kinase. In *B. subtilis*, it was shown that YvcK phosphorylation is irrelevant to growth in restrictive medium but is involved in bacitracin sensitivity^12^. In *L. monocytogenes*, YvcK phosphorylation is unrelated to metabolism and cell wall stress responses, but has a key role in cytosolic survival and virulence^13^. Phosphorylated residues are not conserved across the bacterial species but, in these three bacteria, they are all located in the C-terminus part of YvcK. In *B. subtilis*, we can imagine that the phosphorylation of Thr304, that is located near the Arg301 in the sequence and in the structure, modifies YvcK ability to bind UDP-sugars and thus the bacitracin sensitivity of Bacillus cells.

## Conclusion

In this paper we showed that YvcK is a UDP-sugar binding protein whose gene deletion affects cell size. The molecular mechanism by which a *yvcK* deletion influences cell size is unknown but it will continue to be explored in the future. Altogether, our data suggest that YvcK’s intracellular role is complex. It is a protein with at least two cellular roles that can be dissociated by inactivation of the UDP-sugar binding site or of the phosphorylation site. One of YvcK’s function is independent of its ability to bind UDP-sugars (and of its level of phosphorylation) and is involved in the control of cell size and in the regulation of growth and morphology in Krebs cycle intermediates and substrates of the pentose phosphate pathway. The other one is dependent on its ability to bind UDP-sugars (and of its level of phosphorylation) and is associated with bacitracin sensitivity and potentially with PG synthesis.

## Experimental Procedures

### Plasmid and strain constructions

Standard procedures for molecular cloning and cell transformation of *B. subtilis* or *E. coli* were used.

To generate a non-polar markerless *yvcK* deletion mutant, we first replaced the *yvcK* gene by a chloramphenicol gene cassette, carrying out isothermal assembly^31^. For that, the upstream and downstream regions of *yvcK* were amplified by PCR and mixed with a DNA fragment encoding chloramphenicol resistance. The three DNA fragments were assembled into a single big fragment, which was used to transform 168 WT strain. Then, the antibiotic resistance cassette was removed using the plasmid pDR244, available in the Bacillus Genetic Stock Center (http://www.bgsc.org/). The expression of the genes downstream *yvcK* was checked by qRT PCR.

To introduce point mutations in *yvcK* gene in *B. subtilis* chromosome, first we amplified the *yvcK* mutant alleles from pEFK15 to pEFK18, the upstream and downstream regions of *yvcK*, and mixed with the DNA fragment encoding chloramphenicol resistance. For each point mutation, the four DNA fragments were isothermally assembled into a single big fragment, used to transform 168 WT strain. Then, the antibiotic gene cassette was removed using the plasmid pDR244.

In *B. subtilis* strains, *yvcKgfp* gene fusion was expressed from the *P*_xyl_ promoter in the presence of 0.5% xylose. All the strains and plasmids used in this study are listed in Tables 2 and 3, respectively. Primer sequences are available upon request.

**Table 2:** *B. subtilis* strains used in this study.

Strains	Relevant structures or Genotypes	References
168	*trpC2*	Laboratory stock
SG470	*trpC2*, *yvcK*::*cat*	This study
SG471	*trpC2*, *yvcK*Δ*1*	pDR244 → SG470
SG517	*trpC2*, *yvcK*Δ*1*, *amyE*::(*P*_*xyl*_ *yvcKgfp*), *spec*	pEFK6 → SG471
BTM218	*ugtP::erm*	Laboratory stock
SG571	*ugtP*Δ*1*	pDR244 → BTM218
BKE35670	*gtaB::erm*	(Koo *et al*., unpublished) BGSC*
SG567	*trpC2*, *yvcK*::*cm*, *ugtP::erm*	BTM218 → SG470
SG561	*trpC2*, *yvcK*Δ*1*, *ugtP*Δ*1*	pDR244 → SG567
SG562	*trpC2*, *yvcK*Δ*1*, *gtaB::erm*	BKE35670 → SG471
SG520	*trpC2, yvcK T14A*	This study
SG521	*trpC2*, *yvcK N218A*	This study
SG522	*trpC2*, *yvcK Y265A*	This study
SG523	*trpC2*, *yvcK R301A*	This study

**Table 3:** Plasmids used in this study.

Plasmids	Characteristic or Sources
pEFK6	pSG1154-YvcKGFP^4^
pEFK14	pQE30-YvcK^12^
pEFK15	pQE30-YvcK-T14A (this study)
pEFK16	pQE30-YvcK-N218A (this study)
pEFK17	pQE30-YvcK-Y265A (this study)
pEFK18	pQE30- YvcK-R301A (this study)
pDR244	Koo *et al*., unpublished (Bacillus Genetic Stock Center)

### Site-directed Mutagenesis

Point mutations were introduced into the gene by site-directed mutagenesis by PCR amplification of the whole pEFK6 plasmid. The resulting constructs were verified by DNA sequencing.

### Growth tests

*B. subtilis* strains were grown in LB and CE-minimal medium as previously described^3^.

### Expression and Purification of YvcK Proteins

*E. coli* AD494 was transformed with PQE30-YvcK (wild type or mutated allele) and the resulting strains were used for expression and purification of His_6_-tagged YvcK with NTA resin (Qiagen) as described in refs 12, 32.

### Limited proteolysis

For each 20 μl sample, 5 μg of YvcK were pre-incubated for 10 mn at 37 °C with 40 mM NaCl, 1 mM MgCl_2_, 10 mM Tris/HCl, pH 8.0 in the absence or in the presence of UDP-GlcNAc (1 mM). After addition of 0.005 μg of Trypsin (Promega), the reaction mixture was incubated for 0, 5, 10 or 20 min at 37 °C. The digestion was stopped by adding an equal volume of electrophoresis loading buffer to the assay mixtures and by heating 5 min at 100 °C before applying the samples onto a 12.5% sodium dodecyl sulfate polyacrylamide gel electrophoresis.

### Thermal Shift Assay (TSA)

In thin-walled 96-well PCR plates, each well (20 μl) contained 10 μM of YvcK protein and 2 μl of the fluorescent SYPRO^®^ Orange dye solution (Molecular Probes, 5000x, diluted to 100x in water), in 40 mM NaCl, 1 mM MgCl_2_, 10 mM Tris/HCl, pH 8.0 and was heated from 25 °C to 65 °C in 0.5 °C steps. The fluorescence intensity (Ex/Em = 470/570 nm) of SYPRO^®^ Orange was monitored using a real-time PCR apparatus CFX96 (Bio-Rad). The fluorescence of SYPRO^®^ Orange dye changes when it interacts with the protein undergoing thermal unfolding. The denaturation temperature (T_m_) was analyzed from the melt peak using CFX Manager software (Bio-Rad). The shift of T_m_ (ΔT_m_) induced by the presence of ligand was plotted against the concentration of ligand. Curve fitting was performed by using Microcal Origin 5.0 software using the following equation y = ΔT_m_ max * x^n^/(apparent *K*_D_^n^ + x^n^), were n is the cooperative binding site.

### Western blot

The cells were grown at 37 °C in LB medium to OD_600_ = 1 then 1 ml of culture was centrifuged for 1 min at 14000 rpm. Cell pellets were resuspended in 1/10 volume of lysis buffer containing 50 mM HEPES pH 8.0, 200 mM NaCl, 1 mM DTT, 1 mM MgCl_2_, 1 mM CaCl_2_, 1 mM PMSF, 25 U/ml benzonase and 0.6 mg/ml lyzozyme. Extracts were incubated for 30 min at 37 °C then 0.5% SDS were added at room temperature for 30 min. Samples were afterwards heated at 100 °C for 10 min. Samples were run on a 12.5% SDS-PAGE and transferred to nitrocellulose membrane by electroblotting. The membrane was blocked with phosphate buffer saline (PBS) solution containing 5% milk powder (w/v), for 2 h at room temperature with shaking. Then, the membrane was incubated either with anti-GFP antibody (AgroBio) used at 1/10000 dilution and the secondary antibody, a goat anti-rabbit IgG-HRP (Thermo Fisher) used at 1/2000 or with anti-YvcK antibody used at 1/2500 dilution and the secondary antibody, a goat anti-rabbit IgG-HRP (Thermo Fisher) used at 1/2000. After three washes, the Blot membrane was reading by the LAS4000 mini Imager (by chemiluminescence).

### Microscopy

Strains were grown in LB medium or in minimal medium at 37 °C. Live cells were analyzed by microscopy on a Zeiss Upright Axio Imager M2 microscope as described previously^12^ and stained for membrane using FM1-43 (Invitrogen) where necessary. Cell length was calculated as the distance between adjacent septa.

### Measurement of bacitracin resistance

The antibiotic concentration giving 50% growth inhibition (IC_50_) was determined using the microplate assay described previously^33^.

## Electronic supplementary material

Supplementary data
