# Supplementary material for: YvcK, a protein required for cell wall integrity and optimal carbon source utilization, binds uridine diphosphate-sugars
Source: Sci Rep. 2017 Jun 23;7:4139. doi: 10.1038/s41598-017-04064-2 (PMC5482804; doi:10.1038/s41598-017-04064-2)

**YvcK, a protein required for cell wall integrity and**

**optimal carbon source utilization, binds uridine diphosphate-sugars**

Elodie Foulquier and Anne Galinier^*^

Laboratoire de Chimie Bactérienne, CNRS - Aix Marseille University, IMM, 31 Chemin Joseph Aiguier, 13402 Marseille Cedex 20, France.

Gel used to construct Fig 1A:


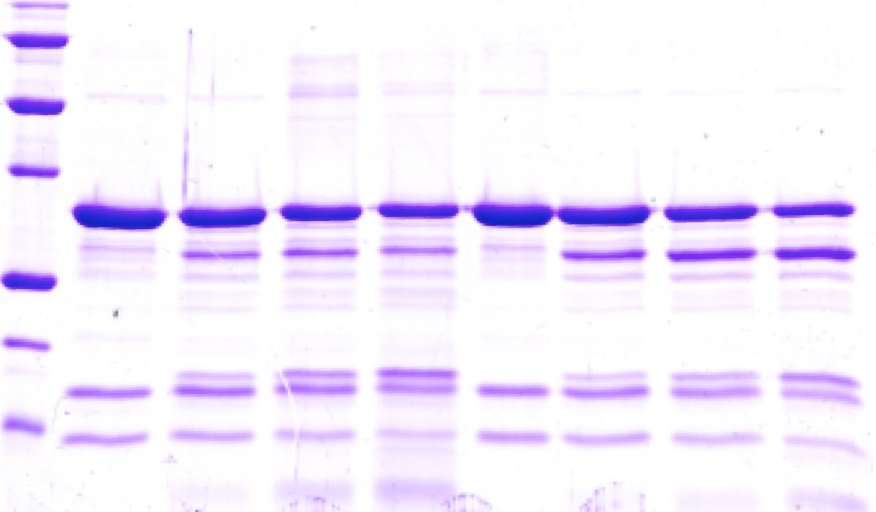


Blot used to construct Fig. 2D (the 3 first bands correspond to the same experiment in absence of xylose used as inductor)

Part used for the manuscript


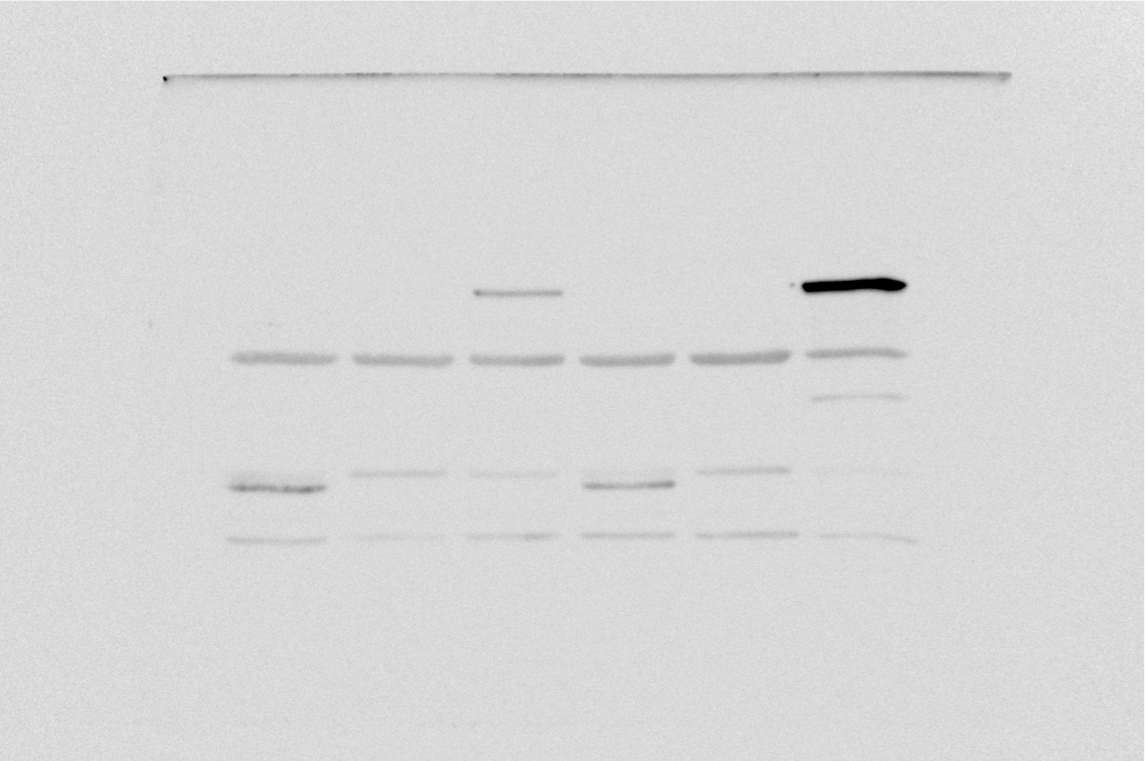


Blot used to construct Fig. 5A (the last lane was not used : it is for a mutant that is not mentioned in this manuscript).

Part used for the manuscript


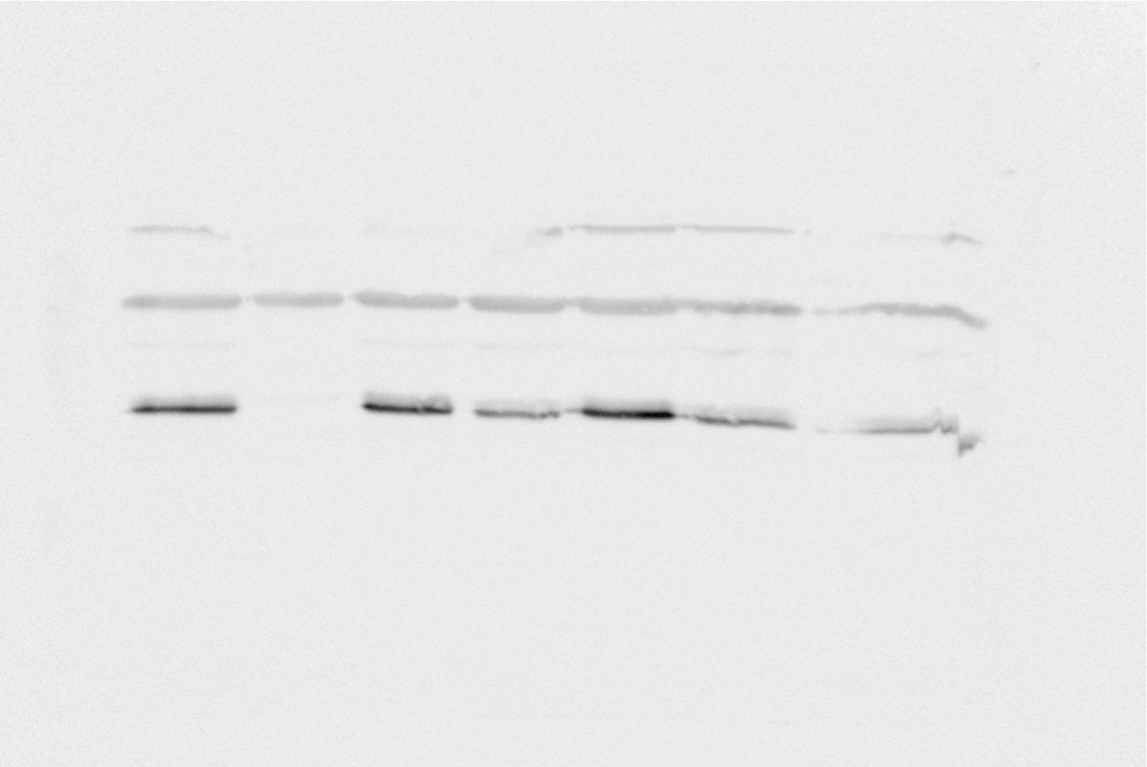

Supplement: Supplementary file 1 — Supplementary data [file 41598_2017_4064_MOESM1_ESM.docx]
